# The complete mitochondrial genome of the brown pansy butterfly, *Junonia stygia* (Aurivillius, 1894), (Insecta: Lepidoptera: Nymphalidae)

**DOI:** 10.1080/23802359.2019.1693921

**Published:** 2019-12-09

**Authors:** Living Prairie Mitogenomics Consortium, Seun Ajibola, Vibhuti Arya, Emily N. Barker, Kirsten T. Biggar, Dominic M. Bohemier, Julina N. Braga, Jessica L. Buchel, Vicky Bui, Julian M. Burtniak, Codey E. Dueck, Steven J. Dupas, Shayna J. Giesbrecht, Alexandra Haverstick, Stefan B. Hreno, Amy L. Irvine, Carter Johnson, Ivory C. Jorgenson, Matthew R. Kroeker, Corrine M. Kuo, Joohee Lee, Vatineh N. Magaji, Gillian J. McIvor, Katrina S. Melgarejo, Michael D. Moore, Olamide U. Ogungbola, Josephine E. Payment, Daniel O. Peter-Salawu, Ashton P. Raitt, Breann T. Recksiedler, Megan Rodriguez, Rahel B. Sahlemariam, Shabadjot Sandhawalia, Mackenzie A. Sarvis, Megan L. Skakum, Jordan C. Small, Kassandra R. Taverner, Chaltu B. Tesfaye, Lea J. Tessier, Catherine J. Unrau, Natasha G. M. Wadlow, Jeffrey M. Marcus

**Affiliations:** Department of Biological Sciences, University of Manitoba, Winnipeg, Canada

**Keywords:** Illumina sequencing, mitogenomics, inquiry-based learning, Lepidoptera, Nymphalidae

## Abstract

The brown pansy, *Junonia stygia* (Aurivillius, 1894) (Lepidoptera: Nymphalidae), is a widespread West African forest butterfly. Genome skimming by Illumina sequencing allowed assembly of a complete 15,233 bp circular mitogenome from *J. stygia* consisting of 79.5% AT nucleotides. Mitochondrial gene order and composition is identical to other butterfly mitogenomes. *Junonia stygia COX1* features an atypical CGA start codon, while *ATP6,* *COX1*, *COX2, ND4,* and *ND4L* exhibit incomplete stop codons. Phylogenetic reconstruction supports a monophyletic Subfamily Nymphalinae, Tribe Junoniini, and genus *Junonia*. The phylogenetic tree places *Junonia iphita* and *J. stygia* as basal mitogenome lineages sister to the remaining *Junonia* sequences.

The Living Prairie Mitogenomics Consortium is an undergraduate inquiry exercise (Marcus et al. 2010) assembling arthropod mitogenomes for improved DNA-based species identification and phylogenetics (Living Prairie Mitogenomics Consortium 2017, 2018, 2019; Marcus 2018). Student participants analyzed sequence data (further curated by the instructor) for presentation here.

*Junonia* butterflies are important models for studying color pattern evolution and development (Marcus 2019). Molecular phylogenetics suggests *Junonia* originated in Africa (Wahlberg et al. 2005; Kodandaramaiah and Wahlberg 2007; Kodandaramaiah 2009). Omitted from these analyses was *Junonia stygia,* the brown pansy, a widespread West African butterfly found in shady understory of disturbed and second growth forests (Larsen 2005; Nyafwono et al. 2014). Larvae feed on plants in the Acanthaceae (Robinson et al. 2010), while adults feed on nectar and are attracted to rotten fruit, manure, and damp salty soil (Molleman et al. 2006; Martins and Collins 2016). *Junonia stygia* is a closed-wing leaf mimic (Suzuki et al. 2014), while dorsally the wings are dark brown and possibly aposematic. Anecdotally, chameleons readily prey upon other *Junonia* but avoid consuming *J. stygia* (Larsen 2006). Most *Junonia* have karyotypes of *N* = 31, but *J. stygia* possess the unusual karyotype of *N* = 33 (Robinson 1971). Here, we describe the complete mitogenome of *J. stygia.*

A leg was removed for DNA isolation (McCullagh and Marcus 2015) and Illumina MiSeq sequencing (Peters and Marcus 2017) from an adult *J. stygia* (Jsty2015.1) collected in Central African Republic in March 2015. The specimen was pinned, spread, and deposited in the Wallis Roughley Museum of Entomology at the University of Manitoba (voucher WRME0501628). The *J. stygia* mitogenome (GenBank MN623383) was assembled with Geneious 10.1.2 from 3,927,312 paired 300-bp reads using a *Junonia lemonias* reference mitogenome (KP41756, McCullagh and Marcus 2015). Annotation was in reference to *J. lemonias*, *Precis andremiaja* (MH917706, Lalonde and Marcus 2019b), and *Salamis anteva* (MH917707, Lalonde and Marcus 2019a). tRNAs were placed using ARWEN v.1.2 (Laslett and Canback 2008). Similarly, the *J. stygia* nuclear rRNA repeat (MN623382) was assembled and annotated using *P. andremiaja* (MH917708) and *S. anteva* (MH917709) reference sequences.

The circular 15,233 bp *J. stygia* mitogenome assembly was derived from 8625 paired reads with nucleotide composition: 39.7% A, 12.8% C, 7.7% G, and 39.8% T. Gene order is identical to other butterfly mitogenomes (McCullagh and Marcus 2015). Three protein-coding genes begin with rare start codons (ATC: *ND3, ND6* (Crozier and Crozier 1993); *CGA: COI* (Liao et al. 2010)), and five genes have single-nucleotide (T: *COX1*, *COX2*) or two-nucleotide (TA: *ATP6*, *ND4*, *ND4L*) stop codons completed by post-transcriptional addition of 3’ A residues. The structure and arrangement of tRNAs, rRNAs, and control region are typical for Lepidoptera (McCullagh and Marcus 2015).

The *J. stygia* mitogenome and 31 other Nymphalid mitogenomes were aligned in CLUSTAL Omega (Sievers et al. 2011), and then analyzed by maximum likelihood and parsimony in PAUP* 4.0b8/4.0d78 (Swofford 2002) (Figure 1). Phylogenetic analysis places *Junonia iphita* and *J. stygia* as basal mitogenomes, sister to the remaining *Junonia* lineages.

**Figure 1.**
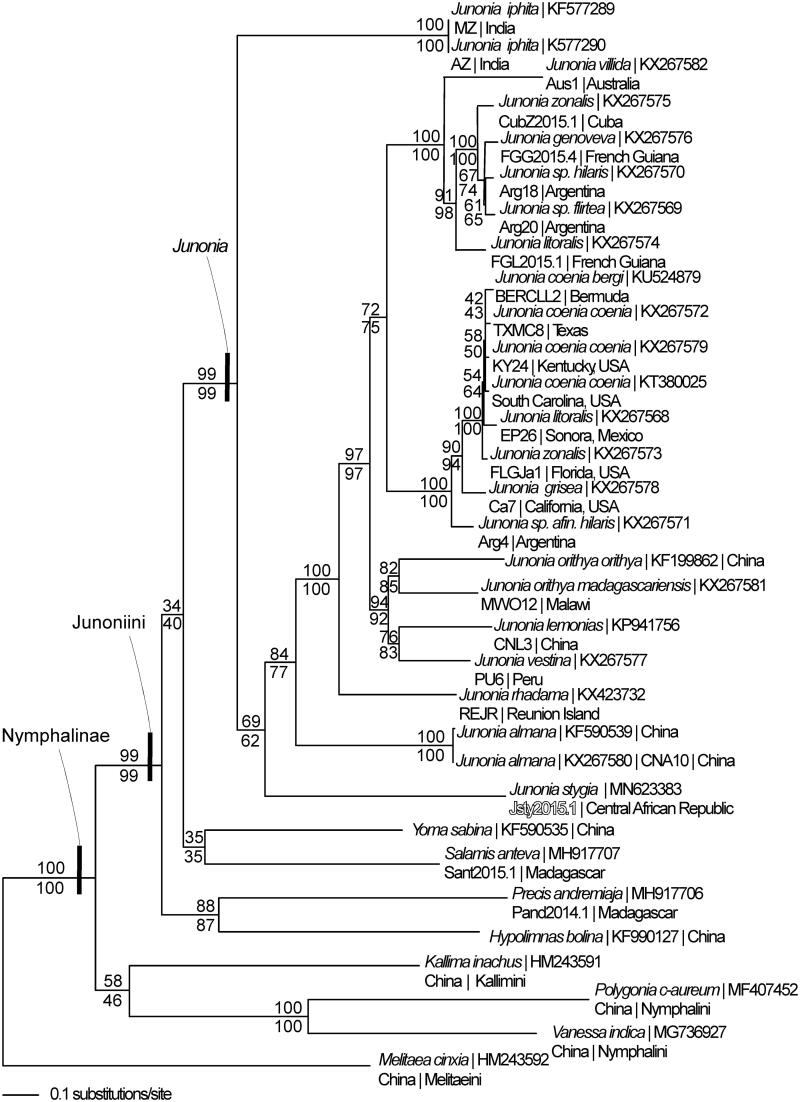
Maximum-likelihood phylogeny of *Junonia* mitogenomes (GTR + G model, G = 0.1940, likelihood score 81636.20542) based on 1 million random addition heuristic search replicates with tree bisection and reconnection. One million maximum parsimony heuristic search replicates also produced eight trees (12,645 steps), one of which was identical to the ML tree, while the others differed only in the arrangement of *Junonia coenia* mitogenomes. Maximum-likelihood (above) and maximum parsimony (below) bootstrap values, each calculated from 1 million random fast addition search replicates, are adjacent to each node. Phylogenetic analysis reveals monophyletic Subfamily Nymphalinae, Tribe Junoniini, and genus *Junonia*.
